# Rejuvenation: an integrated approach to regenerative medicine

**DOI:** 10.1186/2050-490X-1-7

**Published:** 2013-12-02

**Authors:** Y James Kang, Lily Zheng

**Affiliations:** Regenerative Medicine Research Center, West China Hospital, Sichuan University, Chengdu, Sichuan 610041 P.R. China; Department of Pharmacology and Toxicology, University of Louisville, Louisville, KY 40292 USA

**Keywords:** Tissue injury signaling, Repair recruitment, Rejuvenation, Rehabilitation, Aging

## Abstract

The word “rejuvenate” found in the Merriam-Webster dictionary is (1) to make young or youthful again: give new vigor to, and (2) to restore to an original or new state. Regenerative medicine is the process of creating living, functional tissues to repair or replace tissue or organ function lost due to age, disease, damage, or congenital defects. To accomplish this, approaches including transplantation, tissue engineering, cell therapy, and gene therapy are brought into action. These all use exogenously prepared materials to forcefully mend the failed organ. The adaptation of the materials in the host and their integration into the organ are all uncertain. It is a common sense that tissue injury in the younger is easily repaired and the acute injury is healed better and faster. Why does the elder have a diminished capacity of self-repairing, or why does chronic injury cause the loss of the self-repairing capacity? There must be some critical elements that are involved in the repair process, but are suppressed in the elder or under the chronic injury condition. Rejuvenation of the self-repair mechanism would be an ideal solution for functional recovery of the failed organ. To achieve this, it would involve renewal of the injury signaling, reestablishment of the communication and transportation system, recruitment of the materials for repairing, regeneration of the failed organ, and rehabilitation of the renewed organ. It thus would require a comprehensive understanding of developmental biology and a development of new approaches to activate the critical players to rejuvenate the self-repair mechanism in the elder or under chronic injury condition. Efforts focusing on rejuvenation would expect an alternative, if not a better, accomplishment in the regenerative medicine.

## Introduction

Regenerative medicine is an integrated process to recover the organ function lost due to age, disease, damage, or congenital defects [1–7]. The existing approaches for this process include transplantation, tissue engineering, cell therapy, and gene therapy [5, 8–10]. Experimental studies and clinical trials all produce evidence showing the efficacy of these approaches in repairing or replacing the failed organ [5, 6, 8, 10–12]. However, these approaches have a common problem, i.e., the exogenously prepared materials are forcefully applied to the unwilling, failed organ. Thus, extensive efforts have been devoted to the adaptation of the exogenous materials in the host and the functional integration of the materials into the failed organ under repairing. In clinical practice, these involve the life-long immunosuppression of the organ transplantation patients, the creation of the supporting environment for engineered tissues in the mended organ, the unsolved issues of cell survival and differentiation of the cell-based therapy, and the selection and development of vectors for gene therapy.

The biological system is equipped with a self-repair mechanism [13, 14]. Though some organs (e.g. skeletal muscle and liver) trigger better repair than others (e.g. heart and nerve system) when injured, it is undeniable that self-repair mechanism exists in all the tissues of the body, which is a common observation in experimental studies and clinical practice [13–15]. In the younger and under acute injury condition, this repair is a self-motivated and self-directed programmatic process [16, 17]. However, this process is suppressed in the elder and under chronic injury condition [17–21]. The critical questions are: what cause the suppression? What are the lost critical elements leading to the suppression? Can this suppression be relieved?

It is reasonable to believe that there are answers to these questions. Efforts to find these answers have been made, however, comprehensive understanding remains elusive. Nevertheless, there are clues that are noticeable in current undertaking of systems biology and regenerative medicine research. Recruitment of bone marrow cells to the remote injured site is often documented in experimental studies and clinical observations [22, 23]. This indicates that there are signaling systems initiated from the injured site for communicating the injury with the remote repairing mechanism, although these systems have not been recognized. These tissue injury signaling systems would have injury location and potency specificity; helping the repair mechanism recognize the site of the injured organ. They would also share a common pathway for mobilizing the common repair mechanism such as the mobilization of bone marrow cells. The communication between the site of injury and the mobilized repairing materials requires an ensured transportation, vascular and/or lymph system. A damage to any of these signaling, communication, and transportation systems would result in suppression of the self-repair of the injured organ.

Rejuvenation of the self-repair mechanism in the elder or under the chronic injury condition would provide an alternative approach to regenerative medicine. This approach, in contrast to the exogenously prepared repair materials, focuses on mobilization of the self-directed and active rather than passive repair of the injured organ. In this context, the rejuvenation would involve identification and renewal of the tissue injury signaling molecules or pathways. The signaling molecules require a well-connected network, which is different from the intracellular signaling transduction system and demands well-maintained vascular and/or lymph transportation systems. The recruitment of the repair mechanism such as bone marrow cells, and the regeneration of the failed organ are fundamental processes responsible for the effective self-repair. The repair of the structural injury only becomes meaningful if the recovery of the physiological function occurs. Therefore, rehabilitation of the renewed organ is required for the eventual regeneration.

In this commentary, we will consolidate evidence that shows the possibility of rejuvenation of the self-repair mechanism in the elder or under the chronic injury condition, and discuss the elements that are required for the rejuvenation. If rejuvenation of the self-repair or self-renewal mechanism can be developed, an alternative, if not a better, achievement of the regenerative medicine would be expected.

### Renewal of the tissue injury signaling

The tissue injury signaling system should exist in the mammalian system, which would be an active and rescue-requiring signaling system generated from the site of the injured organ and transduced to communicate with the remote repair mechanism. This signaling system is different from that operates in the cell, which is a passive response to the trigger outside of the cell and is generated from the cell membrane, transducing to the inside of the cell. The tissue injury signaling system would ensure the rescue to take place when an injury occurs in any organ in the body. A good example of this system would be the immune response to an injury to an organ. In this case, the signaling molecules would include chemokines and cytokines instantly released from the injured site. It appears that the nature and the severity of the injury dictate the composition and the quantity of the signaling molecules, so that the immune system shows from minor to severe responses.

Ischemic tissue injury occurs in any organ in the body either acutely or chronically. Acute ischemic injury often causes reversible tissue damage and recoverable functional alteration [24–26]. Prolonged and severe ischemic injury results in organ structural deformation and functional loss. Hypoxia-inducible factor (HIF) is responsive to the ischemic injury, and accumulates in the hypoxic cells, leading to up-regulation of multiple genes involved in the response to the injury. Among the genes regulated by HIF are those involved in the vasculogenesis, such as vascular endothelial growth factor (VEGF), placental growth factor (PLGF), angiopoietin 1 (ANGPT1) and 2 (ANGPT2), and Fms-like tyrosine kinase-1 (Flt-1). Other products involved in the mobilization of bone marrow stem cells such as stromal cell-derived factor-1 (SDF-1) and monocyte chemoattractant protein 1 (MCP-1) are also regulated by HIF. These gene products are produced at the injured site and released to the environment; either functioning locally or traveling to the remote sites such as bone marrow. These molecules that are generated in response to tissue injury and are traveling to the remote sites for action would be the composition of the tissue injury signaling system.

It was observed in both experimental studies and clinical investigations that under a long term ischemic condition, these molecules mentioned above are suppressed although severe ischemic condition persists [27–30]. Why does this happen? A series of studies have found at least one answer [31–33]. Under severe ischemic condition, copper ions are depleted in the affected cells [31]. Copper is required for the transcriptional activation of HIF transcription factor [32]. Therefore, even under the condition of HIF protein levels are elevated [34, 35], the up-regulation of the HIF-controlled genes does not occur [31, 32], so that vasculogenesis and the recruitment of bone marrow cells to the injured tissue would not take place. Supplementation with the critical element for HIF transcriptional activity, copper, showed an effective rescue for the HIF activity, the tissue injury communication system, and the repair mechanism [33].

In this ischemic injury example, it appears that hypoxia triggers the response of the injury tissue in the mode of HIF accumulation, which in turn requires cofactors to form a transcriptional complex leading to up-regulation of genes involved in the communication with the remote repair mechanism for the recruitment of repair materials and process. This is a complex process involving multiple factors, thus a missing of any of the critical factors such as copper in its transcriptional activation will lead to the cessation of the communication. In this context, supplementation with the lost factor such as copper as an example helped renew the tissue injury signaling system [31–33].

### Reestablishment of the communication and transportation system

The tissue injury signaling transduction should require the integrity of the signaling molecules and the transduction pathways, as the same as the intracellular signaling system does in the cell. However, an additional requirement is observed in the tissue injury signaling system; transportation system for the remote communication. In case of tissue injury, it is inevitable that the vascular system in the injured tissue is damaged [36]. This damage is often observed in ischemic tissues in the form of infarction; involving cell death and scar tissue formation [37]. This obviously involves degeneration and deformation of the vascular tissue.

Reestablishment of the tissue injury signaling communication thus involves the repair of the damaged vascular system. Not only that the failed transportation system causes problems (e.g. myocardial infarction), it is also easy to understand that the blockage of the circulation system due to the vascular damage is the major obscure of the tissue injury signaling communication. In the intracellular signaling transduction, a missing element in one pathway may lead to the opening and operation of an alternate shuttle pathway. The changes in response to a common trigger but governed by different pathways vary from one condition to another in the cell, but take place one way or the other. However, in the case of tissue injury, the blockage of the circulation system leads to cessation of the remote communication even when all of the essential elements for the communication remain in place. Under this condition, a puzzling situation is often observed that the rescue signaling molecules and the repair mechanism remain intact, but the rescue action fails to take place.

The involvement of the integrity of the circulation system in the communication between the injured site and the remote repair mechanism is a common sense, but is often ignored in the reasoning for tissue response to injury. For instance, in the analysis of myocardial ischemic injury, it was found that VEGF levels were not lower than normal and SDF-1 remained unchanged, but the repair did not take place; inhibition of angiogenesis and myocardial infarction occurred [38–40]. It has been identified that localized progenitor cells or differentiated cells are activated in response to tissue injury and participate in the repair activity [41–44], and the bone marrow cells were shown to be less heroic than expected [45–47]. However, the contribution that bone marrow cells to the repair of tissue injury should not be neglected [6, 11, 48–50].

The transportation system not only ensures the remote communication between the injured site and the repair mechanism [48], but also serves as the essential conduit for the delivery of the repairing materials to the injured site. In the study of myocardial ischemic injury, it was found that intravenous injection of bone marrow stem cells improved the recovery of acute ischemic infarction, but the same treatment had no effect on the chronic ischemic infarction [51–54]. In the latter case, the homing of labeled stem cells was not identified in the infarcted area, otherwise, it took place in the acute infarction [51]. Although the analysis of vascular damage and circulation blockage was not done in the studies cited above, there is no doubt that the infarction involves the vascular injury. Therefore, it is most likely that the circulation blockage would make a significant contribution to the irresponsibility of the infarcted area to the cell therapy.

Reestablishment of the transportation system and the communication pathways for tissue injury signaling transduction obviously plays an important role in the rescue of injured organ. It is easy to understand the importance of the transportation system in the tissue repair, but it is often ignored in the study of tissue repair. A comprehensive understanding of the role of circulation integrity in the tissue injury signaling transduction would make a significant contribution to the rejuvenation of the self-repair mechanism.

### Recruitment of the materials for repairing

What are the materials for the use of tissue repair? Some efforts have focused on bone marrow stem cells in an attempt to treat multiple organ failures and to promote regeneration. On average, bone marrow constitutes 4% of the total body mass of humans; the hematopoietic compartment of bone marrow produces approximately 500 billion blood cells per day, which use the bone marrow vasculature as a conduit to the body's systemic circulation [55]. Bone marrow is also a key component of the lymphatic system, producing lymphocytes that support the body's immune system [56]. Besides these routine activities of bone marrow, the recruitment of bone marrow stem cells in response to tissue injury was one of the major topics of current undertaking of regenerative medicine [49, 50].

Progenitor cells have been considered as a major source of tissue repair or regeneration in response to tissue injury [57, 58]. A progenitor cell, like a stem cell, has a tendency to differentiate into a specific type of cell, but is already more specific than a stem cell and is pushed to differentiate into its "target" cell. The most important difference between stem cells and progenitor cells is that stem cells can replicate indefinitely, whereas progenitor cells can divide only a limited number of times.

Most progenitor cells are described as oligopotent [59, 60]. Therefore, they may be compared to adult stem cells. But progenitor cells are said to be in a further stage of cell differentiation. They are in the “center” between stem cells and fully differentiated cells. The kind of potency they have depends on the type of their "parent" stem cells and also on their niche. Some progenitor cells were found to move through the body and migrate towards the tissue where they are needed. In this context, many properties of adult stem cells are shared by progenitor cells [61]. However, the adult stem cells are quite different from embryonic stem cells that are true stem cells; they are pluripotent and show unlimited capacity for self-renewal. In contrast, it has not been comprehensively demonstrated for the adult stem cells of their capacities for unlimited self-renewal and plasticity [62].

Progenitor cells are found in adult organisms and they act as a repair system for the body. They replenish specialized cells, but also maintain the blood, skin and intestinal tissues [63–66]. These cells often remain dormant or possess little activity in the tissue in which they reside; exhibiting slow growth [65, 67]. The main role of these cells is to replace cells lost by normal attrition [55]. In case of tissue injury, progenitor cells can be activated [67–69]. Growth factors and cytokines are two substances that trigger the progenitor cells to be mobilized toward the damaged tissue [27]. At the same time, they start to differentiate into the target cells. Not all progenitor cells are mobile and are situated near the tissue of their target differentiation. When the cytokines, growth factors and other cell division enhancing stimulators take on the progenitor cells, a higher rate of cell division is introduced, leading to the recovery of the tissue.

Mobilization of bone marrow stem cells has been shown to improve tissue repair after injury [49, 50]. Stem cells released from the bone marrow can migrate into the injured tissue, supporting the process of tissue repair [23, 70]. Efforts have been devoted to the enhancement of bone marrow stem cell mobilization because it has been shown that the number of circulating stem cells is a critical factor for the participation of bone marrow stem cells in tissue repair [71, 72]. In a number of studies addressing various health conditions, higher numbers of circulating stem cells have been associated with greater health. Cytokines, growth factors, and chemokines released from injured tissues are all indicated in the mobilization of bone marrow stem cells [28]. These factors should be considered as the components of tissue injury signaling transduction. They possess dual roles; communicating the injured site with the remote storage of repair materials such as bone marrow stem cells and mobilizing the repair materials for mending action.

The recruitment of repair materials thus involves the mobilization of progenitor cells residing in peripheral tissues and bone marrow stem cells. These materials may be recruited in response to the same set of recruiters or factors sent from the injured site. The extent of the tissue injury and the amount of recruiting factors released to the circulating system may determine the constitution of different repair materials in action.

### Regeneration of the failed organ

Regeneration of the failed organ would be different from repair of the injured organ in many aspects. The repair of injured organs or wounds is one of the most complex biological processes that occur in human life. After an injury, multiple biological pathways immediately become activated and are synchronized to respond. This process involves both local responses and the recruitment of remote repair mechanisms. This repair process leads to different results between human adults and early stage of life. In human adults, a non-functioning mass of fibrotic tissue known as a scar is often observed at the completion of the repair process. By contrast, early in gestation, injured fetal tissues can be completely recreated without fibrosis, which is the process of regeneration.

Regeneration means the regrowth of a damaged or missing organ part from the remaining tissue. Some organs retain high ability to regenerate throughout adult life, such as the liver. If part of the liver is lost by disease or injury, the liver grows back to its original size, though not necessarily to its original shape. However, many other organs are much less capable of regenerating in the adult life. A goal of regenerative medicine is to find and reactivate the missing elements or the suppressed process of regeneration in adult tissues, which exist early in gestation or remain in some organs in the adult life.

Regenerative strategies include the rearrangement of pre-existing tissue, the use of progenitor cells and adult stem cells, and the dedifferentiation and/or transdifferentiation of cells. Dedifferentiation of cells means that they lose their tissue-specific characteristics as tissue remodels during the regeneration process. Transdifferentiation of cells is when they lose their tissue-specific characteristics during the regeneration process, and then re-differentiate to a different kind of cells. Multiple regulatory mechanisms operate in the regeneration process and distinguishingly function in one tissue type or the other. All these strategies result in the re-establishment of appropriate tissue polarity, structure, and form [13, 14, 41, 42, 73].

In a study understanding the process of kidney damage and regeneration, a molecular regenerative pathway was identified [74]. This pathway involves macrophages that respond to tissue injury by producing Wnt7b. The Wnt7b is important to the formation of kidney tissues during embryonic organ development. In the regeneration of injured kidneys, macrophages, by migrating to the injured kidney and producing Wnt7b, re-establish an early molecular program operating in the organ development that becomes beneficial to tissue regeneration. Wnt7b belongs to Wnt family of proteins, which regulate cell growth, proliferation and differentiation [75, 76]. Wnt proteins are also linked to the regulation of stem cells in bone marrow and skin [76–78].

Either residing around the injured site or being remote at the bone marrow, stem cells are necessary for the regeneration of the failed organ. Therefore, efforts have been made to use stem cells as therapeutic agents to promote tissue regeneration [6, 44, 48, 79]. In this context, multiple types of stem cells have been introduced to the injured organs by direct injection or blood infusion [6, 44, 80]. The success of this approach is limited. In acute tissue injury, direct injection of stem cells to the injured organ demonstrates some promotion effect on regeneration [6, 48]. In chronic tissue injury, the same approach fails to demonstrate the beneficial effect [81]. There are multiple reasons for this distinction, but the lost sensitivity to tissue injury, as discussed above, and suppressed capability of tissue regeneration would be responsible for the refractory response under chronic tissue injury condition.

Cell death is a major drawback in cell injection directly to the injured organ for tissue regeneration. As discussed above, tissue regeneration would be a complex and integrated process. The autonomous regeneration process in response to tissue injury would require the trigger of the process or the tissue injury signaling transduction, the preparation of regenerative environment, and mobilization of the regenerative material supplies. In the process of the injection of stem cells directly to the injured organ, it would be forcing the unwilling tissue to receive the regenerative materials and to passively respond to the regenerative action. Since the environment is not prepared for such an action, this forced regenerative process would fail to demonstrate the effectiveness.

The integration of the injected cells in the mended organ is another important concern of the forced regenerative process. Only when the exogenously added cells become integrated structurally and functionally with the existing tissue would regeneration take place. In the forced regenerative process, the unprepared environment of the mended tissue would refuse this integration.

The regeneration of the failed organ would not only require the regeneration materials to reproduce the lost part, but also demand regeneration-friendly environment to adapt and integrate the material in the existing tissue. Forced regenerative process would only cause passive response of the mended tissue to the regeneration materials, and rejuvenation of the regeneration-demanding milieu would greatly help resume the autonomous regenerative process.

### Rehabilitation of the regenerating organ

The regenerated organ has to adapt the existing homeostasis of the entire body in order to become functionally vital organ. This has been a major task in the organ transplantation in which the transplanted organ is subjected to a series of adapting process to reach its life-resuming potential [82, 83]. Rehabilitation would be also critical in the success of regenerative medicine. In today’s clinical practice, regenerative medicine and rehabilitation coexist as serial processes in patient treatment and care plans. However, it is inevitable that the integration of rehabilitation into regenerative medicine takes place to reach the optimal end point.

The treatment of skeletal muscle has evidenced the integration of regenerative medicine and rehabilitation. The repair of muscle injury, regardless of the cause of injury, has been characterized to consist of degeneration, inflammation, regeneration, and fibrosis [84, 85]. Acute and minor injuries typically heal well, but chronic and severe muscle damage is often healed incompletely and with scar tissue formation or fibrosis. As long as the scar persists, complete muscle regeneration is not possible.

Regenerative medicine has brought an alternative approach toward the treatment of skeletal muscle injuries by promoting myofiber regeneration and inhibiting the formation of scar tissue. Transforming growth factor (TGF)-β1 has been shown to be a responsible factor for scar tissue formation [86, 87], and therefore administration of TGF-β1 specific inhibitors has been proposed as an anti-fibrogenic approach [88–90]. In animal models, the presence of TGF-β1 antagonists has significantly decreased fibrosis while concomitantly improving myofiber regeneration [88, 89].

Myofiber regeneration is largely accomplished by muscle stem, or satellite, cells. These cells are localized to the myofiber periphery and activated in response to muscle injury [91]. In the elders, the age-related dysfunction of these muscle stem cells leads to an impaired healing response following skeletal muscle injury. This takes place because that circulating factors found in the aged microenvironment drive the differentiation of muscle stem cells from a myogenic to a fibrogenic lineage [92], resulting in the enhanced scar tissue formation. The profibrogenic switch is also accompanied by a decreased proliferative capacity of aged muscle stem cells further compromising the regeneration. It is thus a logical proposal to transplant young muscle stem cells as an approach to enhance the regenerative potential of aged skeletal muscle. Unfortunately, this was not proven to be desirable. The transplantation of even embryonic stem cells into an aged milieu shows a rapidly decline in their regenerative potential [93].

Rehabilitation to promote the rejuvenation of the aged skeletal muscle niche would help the success of the transplantation of stem cells for the treatment of skeletal muscle injuries. A rodent study has shown that a treadmill running after stem cell transplantation into severely contused muscle increases the myogenic contribution of the donor cells [94]. Therefore, a synergistic effect between physical therapeutics and cellular therapies may exist. The muscle contractile activity may serve as a powerful tool for rejuvenating the regenerative potential of aged muscle. There is accumulated evidence showing that even exercise programs initiated late in life may enhance the ability of muscle to heal itself after severe injury while concomitantly decelerating tissue degeneration [95–97].

Traditional rehabilitation training programs focus on whole body and physiological responses to mechanical loading and/or modalities. However, regenerative medicine pays more attention to molecular, cellular, and histological aspects of tissue regenerative machineries. The integration of the two approaches is a great challenge, but would foreseeably generate a synergetic impact on tissue regeneration.

### Perspectives

Rejuvenation of the self-regeneration mechanism would be an ideal solution for functional recovery of the failed organ. To achieve this, it would involve renewal of the injury signaling, reestablishment of the communication and transportation system, recruitment of the materials for regeneration, regeneration of the failed organ, and rehabilitation of the regenerating organ. It thus would require a comprehensive understanding of developmental biology and development of new approaches to activate the critical players to rejuvenate the self-repair mechanism in the elder or under chronic injury condition. Efforts focusing on rejuvenation would expect an alternative, if not a better, accomplishment in the regenerative medicine.
